# Bribery, New Brunswick and Tobacco

**Published:** 1854-04

**Authors:** 


### BRIBERY, NEW BRUNSWICK AND TOBACCO.

It is stated that a gentleman offers fifty dollars worth of books, to any theological Student in the Seminary at New Brunswick, New Jersey, who will immediately abstain from the use of tobacco in all its forms, and promise to do so for life. I venture to say that the young man who can only be hired from a vice is a most unfit clerical material. If the use of tobacco is not mischievous, it is wrong to hire a fellow-creature to abstain from a harmless gratification: if its use is hurtful, destroying health, constitution, mind and body, which, by the way, is its demonstrable tendency, then, any noble minded young man will abandon its use, because of its pernicious tendency, and will scorn to receive pay for doing what is right. Many who use tobacco, think that it does them no injury, and some contend that it is a benefit. So does the drunkard. A conscientious man will not rest a practice on a bare impression, especially when that impression is contrary to the convictions of older and wiser men, whose observations and studies have been directed to the point in question for years together. Under such circumstances, the thorough investigation of the subject becomes an imperative duty, and no brave heart will be repelled from that investigation, from the fear that a change of his convictions will deprive him of a vast amount of animal gratification.

A wiser, and more practical philanthropist, has originated a far more efficient plan for promoting the disuse of tobacco. He offered three prizes for three "Essays on Tobacco," as follows :—

1. Tobacco, its History, Nature, and Effects, with Facts and Figures for Tobacco Users.
2. The Evils of Tobacco, as they affect Body, Mind, and Morals.
3. Tobacco Diseases, with a Remedy for the Habit.

The prizes were awarded to R. T. Trall, M.D., for the first; to Rev. Dwight Baldwin, for the second; to Joel Shew, M.D., for the third. These Essays have been published by Fowlers and Wells, 131, Nassau-street, New York, in neat tract form, of some twenty 12mo. pages each. Let these three tracts be placed on the desk of every theological Student in the United States, and let benevolent Christian men forward any amount of money they may feel authorized to do, and the Editor of this Journal will, for his part, give personal attention towards having the thing accomplished *at once.* I think that no man of any mental calibre, unblinded by a habit of animal gratification, can possibly read these tracts and not put his foot on the ground and say instantly, "By the help of God, I'll never use it again."

